# The association between erosive toothwear and asthma – is it significant? A meta-analysis

**DOI:** 10.1038/s41405-023-00137-9

**Published:** 2023-03-01

**Authors:** Gowri Sivaramakrishnan, Kannan Sridharan, Muneera Alsobaiei

**Affiliations:** 1 Specialist Prosthodontist and Dental Tutor, Dental Postgraduate training department, Ministry of Health, Manama, Bahrain; 2 Department of Pharmacology and Therapeutics, College of Medicine and Medical Sciences, Arabian Gulf University, Manama, Bahrain; 3 Acting Head of Training Affairs, Dental Postgraduate training department, Ministry of Health, Manama, Bahrain

**Keywords:** Gum disease, Gingivitis

## Abstract

**Background:**

The association of asthma with oral conditions such as dental caries, dental erosion, periodontal diseases and oral mucosal changes has been the subject of debate among dental practitioners. Existing evidence indicates that an inhaler is the most common and effective way of delivering the asthma medications directly into the lungs. Few studies in the past attributed this association to the changes in salivary flow caused due to these medications. Considering this unclear association, the aim of the present meta-analyses is to identify the association between erosive toothwear and asthma from individual studies conducted until date.

**Methodology:**

Electronic databases were systematically searched until 30th September 2022. Articles identified using the search strategy were imported to RAYYAN systematic review software. Data was extracted relating to study design, geographic location, year of publication, sample size, the assessment method for erosive toothwear and asthma. The Newcastle Ottawa scale was utilized to assess the quality of evidence reported from the included studies. RevMan Version 5.3 was used to perform a random-effects meta-analysis to produce pooled estimates from OR and 95% CI of included studies. The I² statistic was used to determine the extent of heterogeneity. A funnel plot was generated to visually assess the potential for publication bias. Sensitivity analyses were performed by excluding individual studies one at a time. GRADE approach was used for grading the evidence for key comparisons.

**Results:**

Twelve articles were included in the final meta-analysis. A total of 1027 asthmatics and 5617 non-asthmatics were included. All studies demonstrated moderate to low risk of bias. The overall pooled estimate (OR: 2.03; 95% CI: 0.96, 4.29) and subgroup analyses in children (OR: 1.67; 95% CI: 0.63, 4.42) did not show statistically significant difference in the occurrence of dental erosion between the asthmatic and non-asthmatic group. However, asthmatic adults had significantly greater dental erosion in comparison to the control adults (OR: 2.76; 95% CI: 1.24, 6.16). Sensitivity analyses also provided inconclusive evidence. Funnel plot asymmetry indicated significant heterogeneity, changes in effect size and selective publication.

**Conclusion:**

The association between inhalational asthmatic medication and tooth wear is inconclusive. There are a number of confounding factors that play a greater role in causing dental erosion in these patients. Dentist must pay particular attention to these factors while treating asthmatic patients. The authors produce a comprehensive checklist in order to ensure complete assessment before providing advice on their medications alone.

## Introduction

Asthma is a chronic airway inflammatory disease that causes increased airway hyperresponsiveness, leading to symptoms such as wheezing, coughing, chest tightness and dyspnoea [1] It is characterized by the obstruction of airflow that varies over a period, and is reversible spontaneously, or with medications [2]. Asthma is a global public health problem and recent estimates reveal that over 400 million people may be diagnosed with asthma by 2025 [2]. The treatment of asthma usually involves prescription of drug classes such as beta-2 agonists, corticosteroids, antimuscarinics, leukotriene inhibitors and xanthines [3]. They primarily act by controlling, as well as to reducing the airway inflammation, and reopen the airways [4]. These medications are used in the form of inhalations, tablets, capsules and injections, etc. The dose and frequency of the medications is dependent upon the severity of the disease and its related symptoms [5].

Inhalers are devices that deliver the medication directly into the airway through the mouth. Majority of the asthma patients use various forms of inhalers that are prescribed for use for upto three times daily [6]. Inhalation preparations include solutions for nebulization, metered-dose inhalers, and powdered inhalers [7]. Existing evidence indicates that an inhaler is the most common and effective way of delivering the asthma medications directly into the lungs [8]. However, each inhaler is unique in their mechanism of action. Some inhalers emit an aerosol jet when activated. They work better in conjunction with a spacer, which is a plastic or metal container, with a mouthpiece at one end and a hole for the inhaler at the other end. The spacer helps in delivering the medication straight into the lungs. This means that there is less medication ending up in the mouth or throat, which has been reported to cause irritation or soreness. Spacer also helps in coordinating breathing in and pressing the puffer [9].

The association of asthma with oral conditions such as dental caries, dental erosion, periodontal diseases and oral mucosal changes has been the subject of debate among dental practitioners [10]. Anti-asthmatic medications specifically the inhalers are always associated with causing dental erosion and toothwear. Dentists that strongly believe in this association between erosive toothwear and asthma suggested the use of spacer or advice other alternative medications to their patients [11]. Few studies in the past attributed this association to the changes in salivary flow caused due to these medications [10, 11]. Reports in the past also showed decreased output of salivary amylase, hexosamine, salivary peroxidase, lysozyme and secretory IgA in stimulated saliva of asthmatic patients [10]. These changes in the quality and quantity of saliva has been linked to toothwear and dental erosion. Inspite of all the available evidences that seem to associate erosion with asthma, majority of the individual studies conducted in the past did not show statistically significant differences in prevalence of erosive toothwear between asthma patients and asthma controls [12, 13]. In addition higher prevalence of toothwear was reported in asthmatic patients with additional confounding factors such as acidic diet, parafunctional habits, gastrointestinal reflux diseases etc. [14]. This is important from the dentist perspective because dentist that strongly believe in the association may not be interested to look at other confounding factors for toothwear. It is extremely important for the dentist to manage the confounding factors as well, and not only focus on asthma and its medications in patients presenting with erosive toothwear. Considering this unclear association, the aim of the present meta-analyses is to identify the association between erosive toothwear and asthma from individual studies conducted until date.

## Material and methodology

### Protocol and registration

This systematic review and meta-analysis was conducted and reported according to PRISMA (Preferred Reporting Guidelines for Systematic Reviews) guidelines [15]. A protocol was developed (CRD42022324844) and submitted to PROSPERO. The protocol can be assessed @ https://www.crd.york.ac.uk/prospero/export_details_pdf.php. The systematic review protocol clearly described the intention to study the bidirectional relationship between erosive toothwear and asthma and its related medications. Since majority of the studies reported the prevalence of erosive toothwear asthmatics, this study is particularly restricted to erosive tooth wear. Since this is a meta-analysis, formal ethics approval is not required for this type of study.

### Data sources and search strategy

Ovid MEDLINE, Scopus, Embase, and Web of Science electronic databases were systematically searched until 30th September 2022. The gray literature was hand searched for records that were not electronically accessible or for those manuscripts without an electronic abstract. Further searches were undertaken to cross check references not available in the electronic databases. The search strategy included synonyms for erosive toothwear combined with synonymous terms for asthma, asthmatic medications and asthma related symptoms. The keywords were searched alone and in combination to retrieve relevant literature. This systematic review included two types of studies: cross-sectional and case-control, which analyzed the association between erosive toothwear and asthma. There were no randomized controlled trials that were obtained during the initial search. The search was limited to humans, adults and publications in the English language.

### Study selection and eligibility criteria

Articles identified using the search strategy was imported to RAYYAN systematic review software [16]. Using this software, duplicates were removed, and two independent reviewers (GS and KS) screened all titles and abstracts. Potentially relevant full-text articles were then read to determine if an article met the inclusion criteria. A discussion between the two reviewers was held to reach an agreement. In order to test agreement between the first and second reviewers, kappa values were calculated at each stage of data extraction.

### Inclusion and exclusion criteria

All human studies performed in either children or adults which included a dental assessment (oral examination) for erosive toothwear using any standardized indices that are available, and asthma disease and medication assessment (medical assessment or self-reported), were eligible for inclusion. Case reports, case series, opinion papers and reviews were excluded.

### Data extraction and study quality assessment

Data was extracted relating to study design, geographic location, year of publication, sample size, the assessment method for erosive toothwear and asthma. The Newcastle Ottawa scale (NOS) was utilized to assess the quality of evidence reported from the included studies [17].

### Statistical analysis

Review Manager (RevMan Version 5.3, The Cochrane Collaboration) was used to perform a random-effects meta-analysis to produce pooled estimates from odds ratios (OR) and 95% confidence intervals (CI) of included studies. The OR and 95% CI were calculated from prevalence data reported in the study. The I² statistic was used to determine the extent of heterogeneity in included studies and values above 50% was considered as substantial heterogeneity [18]. A funnel plot was generated to visually assess the potential for publication bias. Sensitivity analyses were performed by excluding individual studies one at a time with the intention of assessing the robustness of the pooled data. We used grades of recommendation, assessment, development and evaluation (GRADE) approach for grading the evidence for key comparisons.

## Results

**Fig. 1:**
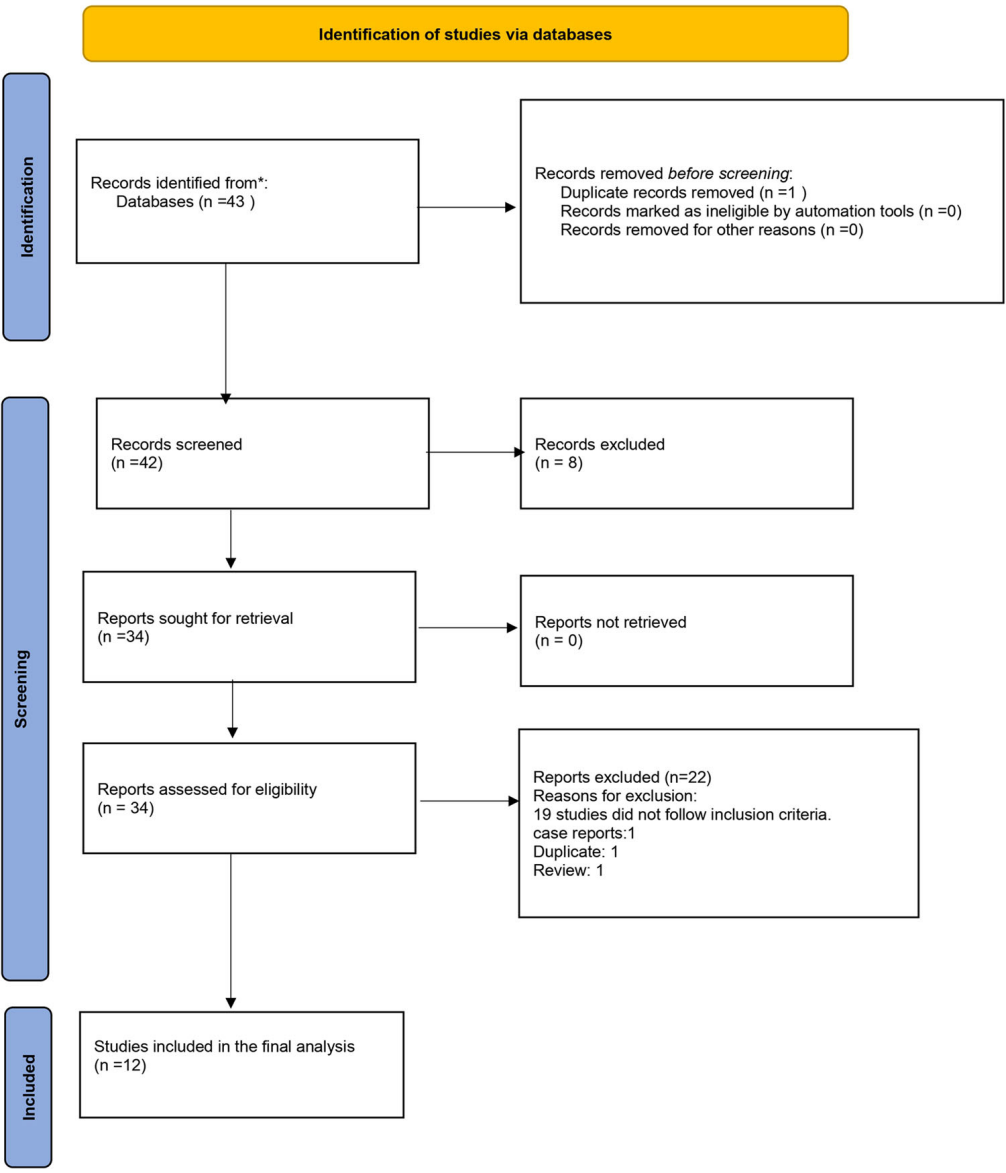
Preferred Reporting Items for Systematic Reviews and Meta-Analyses(PRISMA) flow diagram. An evidence-based minimum set of items for reporting in systematic reviews and meta-analyses.

The initial search strategy identified articles, which was reduced to 42 articles after duplicates were removed. Thirty-four articles met the inclusion criteria for full text assessment. After excluding 22 papers following the full text screening, 12 [12–14, 18–26] articles were included in the final meta-analysis. The detailed search strategy is presented in the PRISMA Flow diagram (Fig. 1).

### Asthma and dental erosion

Eight studies were conducted in children and four studies in adult population. A total of 1027 asthmatics and 5617 non-asthmatics were included in the meta-analysis. 775 (75%) asthmatic patients were taking inhalational anti-asthmatic medications. Majority of these medications were inhalational steroids, bronchodilators like salbutamol or a combination of these. The corticosteroid inhalers included drugs such as budesonide, fluticasone or betamethasone. Four [18, 20, 21, 23] out of the included 12 studies did not mention the details regarding the medication. The asthmatic history was extracted subjectively using questionnaire given to adult patients, or parents of asthmatic children. The erosive toothwear was examined objectively using clinical examination and classified according to indices such as Basic erosive wear examination, Smith and knight index *etc* in all the included studies. The confounding factors that were considered were oral hygiene and brushing habits, acidic food consumption, acidic or soft drinks, saliva quality, gastric disorders and activities like swimming. Not all studies included all the confounding factors mentioned above. Two studies [12, 22] however did not clearly mention the confounders they considered in their patients. Two studies demonstrated that 100% of the participants in the asthmatic and non-asthmatic groups had dental erosion[14, 19]. 61 out of the 64 asthmatic patients in these two studies were on medications. All studies demonstrated moderate to low risk of Bias on eight domains as observed using the New Castle Ottawa Scale (Table 1). The characteristics of included studies is presented in Table 2.

**Table 1:** Risk of bias using New Castle Ottawa scale.

Study Id	Selection				Comparability	Outcome			
	Representativeness of exposed cohort	Selection of the non-exposed cohort from same source as exposed cohort	Ascertainment of exposure	Outcome of interest was not present at start of study	Comparability of cohorts	Assessment of outcome	Follow-up long enough for outcome to occur	Adequacy of follow-up	Quality score
Alazmah [18]	*	*	*	*	*	*	*	*	8
Al-Dlaigan et al. [19]	N	*	*	*	N	*	*	*	6
Alves et al. [20]	N	*	*	*	N	*	*	*	6
Arafa et al. [21]	N	*	*	*	N	*	*	*	6
Dugmore and Rock [12]	N	*	*	*	N	*	*	*	6
Farag and Awood [22]	*	*	*	*	*	*	*	*	8
Jain et al. [25]	*	*	*	*	*	*	*	*	8
Jacob et al. [24]	N	*	*	*	N	*	*	*	6
Gurgel et al. [23]	N	*	*	*	N	*	*	*	6
Rezende et al. [13]	*	*	*	*	*	*	*	*	8
Stensson et al. [26]	*	*	*	*	*	*	*	*	8
Sivasithamparam et al. [14]	N	*	*	*	N	*	*	*	6

**Table 2:** Key characteristics of included studies.

Study id	Location	Age range of study participants (in years)	Asthma			Erosive toothwear		Confounders looked at	Risk of Bias assessment using New castle Ottawa scale
			Diagnosis	Number cases/control	Taking asthma medication	Diagnosis	Outcome in Cases/control		
Alazmah [18]	Saudi Arabia	3–12	Questionnaire	50/50	Not mentioned	Smith and knight index	12/9	Brushing frequency	Low
Al-Dlaigan et al. [19]	UK	11–18	Questionnaire	20/40	20	Smith and knight index	20/40	Heartburn, stomach problems, vomiting	Moderate
Alves et al. [20]	Brazil	12	Interview	118/1410	Not mentioned	Basic erosive wear examination	20/210	Daily consumption of soft drinks, regurgitation, heart burn	Moderate
Arafa et al. [21]	Saudi Arabia	4–12	Questionnaire	60/120	Not mentioned	Smith and knight index	48/8	Saliva flow rate, DMFT	Moderate
Dugmore and Rock [12]	UK	12–14	Questionnaire	479/2582	479	Basic erosive index	294/1787	None	Moderate
Farag and Awooda [22]	Saudi Arabia	18–60	Questionnaire	40/40	40	Basic erosive wear examination	36/35	None	Low
Jain et al. [25]	India	Above 18	Questionnaire	51/51	51	Community periodontal index of treatment needs	39/35	Oral hygiene habit, intake of soft drinks	Low
Jacob et al. [24]	India	Above 12	Questionnaire	19/411	19	Basic erosive wear examination	16/173	GERD, citrus and soft drinks, oral hygiene habits	Moderate
Gurgel et al. [23]	Brazil	12–16	Questionnaire	14/398	Not mentioned	Obrien Index	1/82	Gastric disorders, acidic drinks, activities like swimming	Moderate
Rezende et al. [13]	Brazil	6–12	Questionnaire	112/116	105	Modified Dental examination	36/44	Oral hygiene habits	Low
Stensson et al. [26]	Sweden	18–24	Interview	20/20	20	Johansson et al. grading system	15/8	Oral hygiene, caries status, salivary factors	Low
Sivasithamparam et al. [14]	Australia	15–55	Interview	44/379	41	Scanning electron microscope	44/379	Acid consumption, soft drink consumption, gastric complaints	Moderate

### Subgroup analyses pooled estimates

**Fig. 2:**
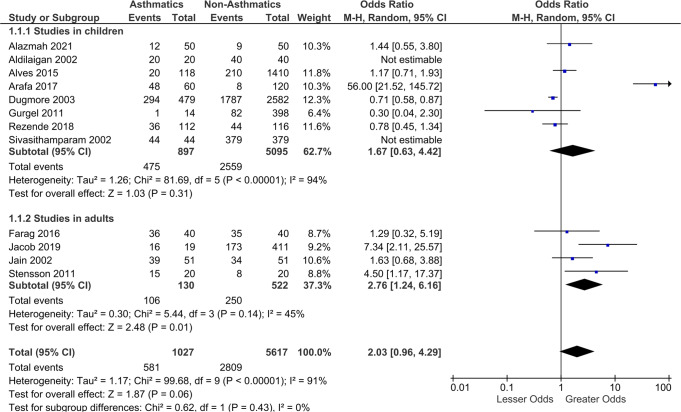
Forest plot. Diagram depicting the association between asthma and dental erosion.

Forest plot was generated using the software and the overall pooled estimate (OR: 2.03; 95% CI: 0.96, 4.29) did not show statistically significant difference in the occurrence of dental erosion between the asthmatic and non-asthmatic group (Fig. 2).

Subgroup analyses was done for studies in children and in adults. Studies in children included 897 asthmatic children and 5095 children in the control group. The pooled estimates in children (OR: 1.67; 95% CI: 0.63, 4.42) did not show statistically significant differences in the presence of dental erosion in asthmatics and non-asthmatics. However, asthmatic adults had significantly greater dental erosion in comparison to the control adults. The pooled estimate was statistically significant (OR: 2.76; 95% CI: 1.24, 6.16).

### Sensitivity analyses

**Fig. 3:**
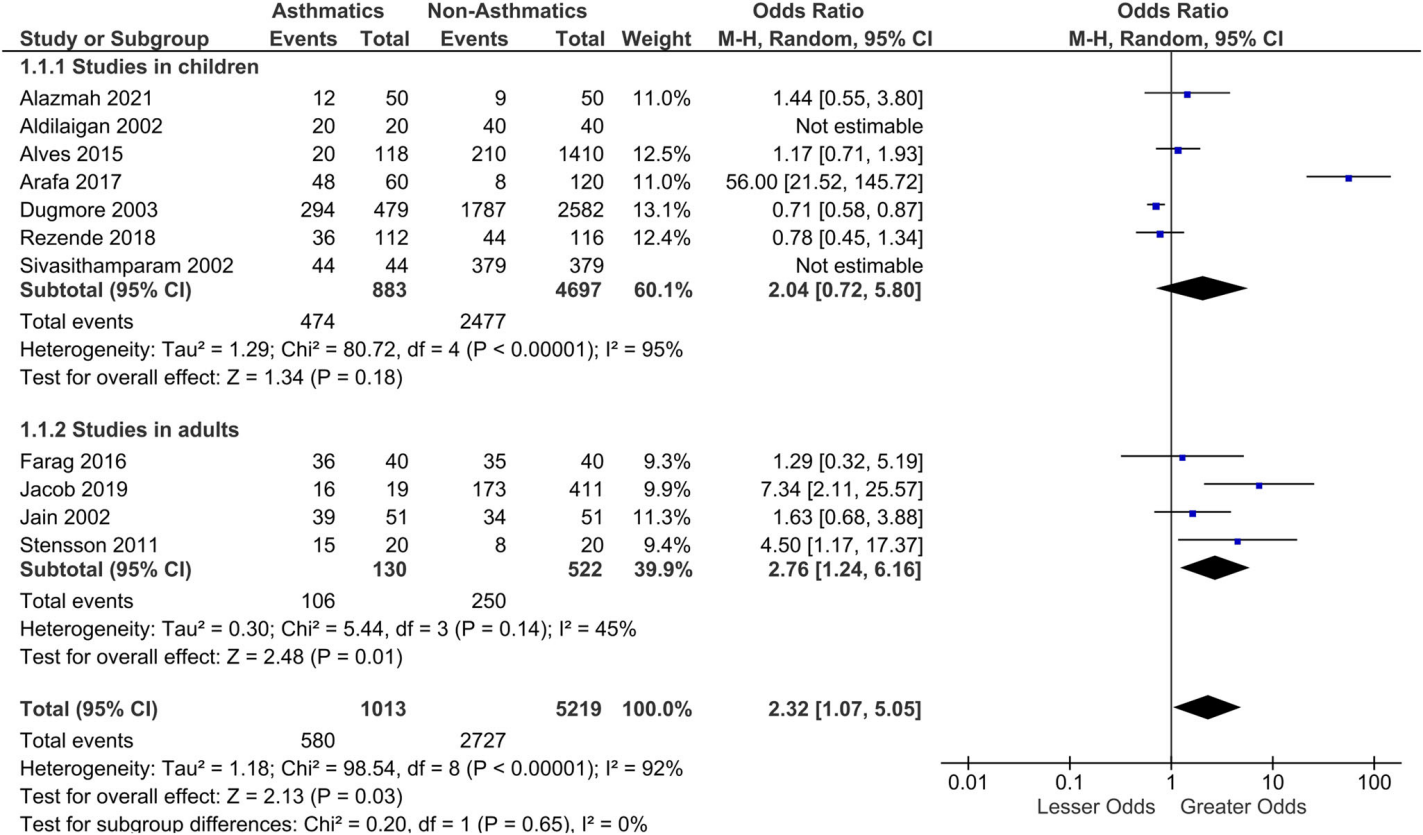
Forest plot. Diagram after removing the study by Grugel et al.

Sensitivity analyses after removing the outlier study [21] identified using visual examination of the forest plot did not show significant difference in the pooled estimate. However, this study was majorly contributing to the heterogeneity as observed by the I^2^ value. Sensitivity analyses removing Grugel et al. study yielded statistically significant pooled estimated (OR: 2.32; 95% CI: 1.07, 5.05) with greater dental erosion in asthmatic group (Fig. 3). The forest plot after removing the study by Grugel et al. is presented in Fig. 3. Studies that did not mention the details regarding the asthmatic medication in children were removed and the pooled estimate showed statistically significant lesser odds of dental erosion in children reported to be on inhalational asthmatic medication (OR: 0.71; 95% CI: 0.59, 0.86). The other significant findings from the sensitivity analyses are presented in Table 3.

**Table 3:** Sensitivity analyses.

Sensitivity analyses	Asthmatic group (*N*)	Non-asthmatic control (*N*)	Pooled estimate at 95% CI	Heterogeneity I2
Overall – Removing Grugel et al.	1013	5219	2.32 [1.07, 5.05]^a^	92%
Overall – Removing Arafa et al. – outlier study	967	5497	1.28 [0.82, 2.02]	70%
Overall – Removing Arafa et al. and Grugel et al.	953	5099	1.37 [0.86, 2.18]	73%
Overall – studies with no confounders removed	508	2995	2.51 [0.92, 6.82]	90%
Overall – studies with moderate ROB removed	233	237	1.43 [0.74, 2.76]	55%
Studies in Adults:				
Removing Stensson et al.	110	502	2.43 [0.88, 6.77]	57%
Studies in Children				
Removing Arafa et al.	837	4975	0.83 [0.63, 1.11]	30%
Studies with no asthmatic medication details removed	655	3117	0.71 [0.59, 0.86]^a^	0%

^a^Statistically significant.

### Funnel plot and publication bias

**Fig. 4:**
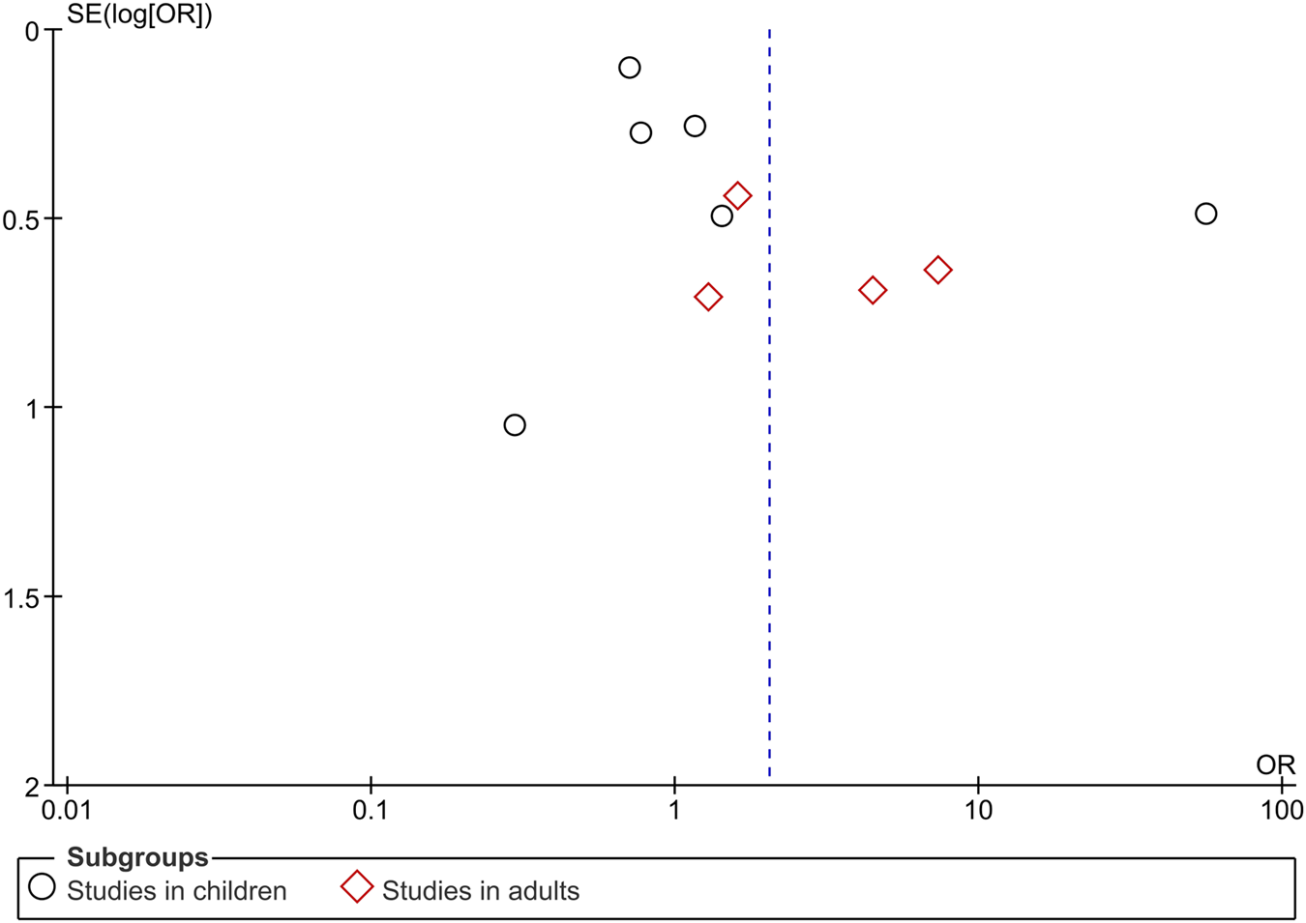
Funnel plot. Diagram depicting publication bias.

The funnel plot depicted in Fig. 4 does not suggest any publication bias in adult population. However, asymmetry was observed in children, probably caused due to significant heterogeneity between the studies. This heterogeneity can be defined as the differences in the size of the effect according to the study size. Majority of the included studies had greater number of patients in the control group. There are also major differences in the underlying confounding factors between the studies. The intervention group had greater differences in the drug, dose, and frequency of the inhaled anti-asthmatic medication. All these factors can lead to the asymmetry as shown in the funnel plot. Asymmetrical funnel plot in studies in children also indicates selective publication of studies according to the results obtained.

### Grading the strength of outcome measure

Grading the strength of outcomes in various study populations is summarized in Table 4. Either a low or very low strength of evidence was observed due to serious limitations in risk of bias/precision of the estimates and publication bias.

**Table 4:** Grading the strength of evidence.

Comparisons	Illustrative comparative risks (per 1000) (95% confidence intervals)		Effect estimates and quality of evidence for mixed treatment comparisons
	Assumed risk^a^	Corresponding risk	
Proportion of patients (adults/children) with dental erosions	500 per 1000	670 (600 to 811)	2.03 [0.96, 4.29] ⊕⊕⊝⊝; Low^b,c^
Proportion of adults with dental erosions	479 per 1000	716 (533 to 849)	2.76 [1.24, 6.16] ⊕⊝⊝⊝; Very low^b,c,d^
Proportion of children with dental erosions	293 per 1000	409 (207 to 647)	1.67 [0.63, 4.42] ⊕⊝⊝⊝; Very low^b,c,d^

Low: Further research is very likely to have an important impact on our confidence in the estimate of effect and is likely to change the estimate; Very low quality: We are very uncertain about the estimate.

^a^Assumed risk was the median control group (non-asthmatics) risk across the studies.

^b^Downgraded one level for including studies with high risk of bias.

^c^Downgraded one level as publication bias could not be assessed/ruled out.

^d^Downgraded one level for serious limitations in the precision of the estimates.

## Discussion

The present meta-analysis is an attempt to identify the association between dental erosion and asthma. This association has been strongly believed by many dentists. The results from the present study does not offer conclusive evidence to point a clear association between anti-asthmatic medications and dental erosion. Although some of the results obtained may show statistical significance, considering the increased heterogeneity in these studies, a conclusive association cannot be elucidated. This is similar to the findings of Moreira et al. [27] however only two studies were included for the analysis.

It is extremely important that dentists understand that there are variety of factors that can contribute to dental erosion such as consumption of acidic pickled fruits and vegetables, frequent intake of citrus juices, fizzy drinks, systemic gastric disorders like gastro esophageal reflux diseases (GERD), bulimia, and frequent vomiting due to various causes. The association between GERD and dental erosion is clearly demonstrated in a recent meta-analysis by Jordao et al. [28]. The study demonstrated that the objectively assessed patients with GERD showed greater odds of erosive toothwear. It is to be noted that the prevalence of GERD is reported to be approximately 75% in patients with asthma [29]. This indicates that all patients that present with dental erosion should be assessed for GERD. Direct aspiration of the gastric contents into the lung tissue stimulates and damages the epithelial cells, leading to the release of inflammatory cytokines. This causes chronic airway inflammation, airway hyperresponsiveness and airway obstruction. Hence, all asthmatic patients, especially those who are obese should be objectively tested for GERD and other gastric disorders. Obesity asthma phenotype is considered as distinct in view of greater severity and poor asthma control [30]. None of the included studies considered this strong association and evaluated their patients for obesity or gastric disorders. It is necessary to understand from the evidence that anti-asthmatic medications are unlikely to be solely responsible for toothwear, and every possible reason that is mentioned above must be excluded.

The most widely used inhaled drugs are corticosteroids, β-adrenergic agonists, and muscarinic antagonists. In patients with persistent asthma, long-term regular use of inhaled corticosteroids is prescribed to achieve asthma control. In severe asthma, additional medications are prescribed to achieve symptom control and prevent exacerbations [30]. The most important feature of these devices is to deliver significant portion of the medication upto the terminal airways to ensure high bronchial deposition. Four main types of inhalers available today are nebulizers, dry powder inhalers (DPIs), pressurized metered-dose inhalers (pMDIs), and soft mist inhalers (SMIs) [31]. Currently, the most popular inhalation device is the DPI, which is considered environment friendly and easy to use [32]. However, it is to be noted that DPI cannot be used with a spacer, which may contribute to dental erosion. In the included studies, clear description of the type of inhaler used by the participants was not provided. This may have an influence on the results obtained. A recent study regarding the ph levels of saliva following inhalation medication reported that all inhalers in the study failed to depress the salivary ph below 6. A substantial pH drop was observed only with the use of lactose-based DPIs, although not below pH 6 [33]. This study suggests that the theory correlating inhalation medication related ph drop and enamel demineralization cannot be justified. Other factors that are mentioned previously play a major role in causing dental erosion in asthmatic patients.

The subgroup analyses conducted in the present study showed contradicting results in children and adults, with adult asthmatics presenting with significant dental erosion. The sensitivity analyses also showed varied results that cannot offer meaningful conclusions. In the overall analyses, the number of participants in the non-asthmatic group outnumbered the comparator group. This has led to significant heterogeneity that is evident from the analyses. The investigators in the included studies did not consider many confounding factors that are discussed previously that has an influence on the outcome. This means that the intervention and comparator groups, in majority of the studies, were ideally non-comparable at baseline. Hence, the results obtained are not conclusive. Although this is the limitation, this meta-analysis identifies that there is a definite need for future studies that should have participants in the treatment and comparator groups that are comparable at baseline. This might possibly help in reducing the heterogeneity and provide conclusive evidence. There were also limited number of studies on adults and children that were available for inclusion that might have influenced the results obtained.

Erosive toothwear occurs because of prolonged exposure of the surfaces of the tooth to acid attacks. Dental treatment strategies are aimed to suggest the use of spacers, alternate medications, specific oral hygiene instructions such as mouth rinsing following inhalation for patients on anti-asthmatic medications. Although it is not inappropriate to provide the above-mentioned instructions to these patients, it is important that the confounding factors also be addressed accordingly. A summarized checklist to be used by dentists when treating asthmatic patients in presented in Table 5. All these factors needs to be considered in order to treat dental erosion in these patients.

**Table 5:** Checklist for dentist when treating patients with asthma.

1	History of asthma – age of onset, frequency, severity
2	Anti-asthmatic medication – drug class, type of inhaler, duration of use, use of spacers, method of use (eg. Swishing the mouth with water after use), compliance with the use of inhalers
3	Body mass index, obesity, Sleep disordered breathing, sleep apnoea
4	Diet- acidic/ pickled food, citrus, fizzy drinks
5	Objective assessment of gastric disorders
6	Local factors – condition of the enamel, hypomineralisation etc

## Conclusion

The association between inhalational asthmatic medication and tooth wear is inconclusive from the results obtained in the present meta-analysis. There are a number of confounding factors that play a greater role in causing dental erosion in these patients. Dentist must pay particular attention to these factors while treating asthmatic patients. The authors produce a comprehensive checklist in order to ensure complete assessment before providing advice on their medications alone. There is a greater need for future studies considering all the factors that are discussed in this paper.

## Supplementary information

Proof of waiver
