# Supplementary material for: The association between erosive toothwear and asthma – is it significant? A meta-analysis
Source: BDJ Open. 2023 Mar 1;9:9. doi: 10.1038/s41405-023-00137-9 (PMC9977957; doi:10.1038/s41405-023-00137-9)
Supplement: Supplementary file 1 — Proof of waiver [file 41405_2023_137_MOESM1_ESM.pdf]

From: Gowri Sivaramakrishnan <gowri.sivaramakrishnan@gmail.com>
Sent: 27 December 2022 11:11
To: Danielle Yates <danielle.yates@nature.com>; Jonathan Lewney <Jonathan.Lewney@bda.org>
Subject: Fwd: A full waiver has been approved

The details are in the trailing mail For your persual.

Thank you
Best regards
Gowri

---------- Forwarded message ---------
From: <do-not-reply@springernature.com>
Date: Wed, 21 Dec 2022, 13:49
Subject: A full waiver has been approved
To: <gowri.sivaramakrishnan@gmail.com>

SPRINGER NATURE

Dear Author,

Many thanks for providing documentation of your funding status.

We have reviewed your request and have approved a full waiver.
If your article is accepted for publication, we will apply the waiver automatically at the payment stage. You do not need to take any further action.

Log in to view the details of your request or contact us:
https://article-discounts-and-waivers.springernature.com/request-summary/e579547c-49f8-4128-8362-9d03e9511f8c

Kind regards,
APC Discount and Waiver Service
